# Nivolumab Plus Ipilimumab for Unresectable Hepatocellular Carcinoma in Early Real-World Clinical Practice: A Multicenter Analysis of Efficacy and Safety

**DOI:** 10.3390/cancers18162703

**Published:** 2026-08-20

**Authors:** Hironao Okubo, Teppei Matsui, Makoto Chuma, Akihiro Funaoka, Haruki Uojima, Satoshi Narahara, Masahiro Kobayashi, Yuwa Ando, Tsunamasa Watanabe, Taito Fukushima, Satoshi Kobayashi, Katsuharu Hirano, Kota Tsuruya, Tatehiro Kagawa, Shuichiro Iwasaki, Hisashi Hidaka, Hidenari Nagai, Yasuhito Tanaka, Shin Maeda, Manabu Morimoto

**Affiliations:** 1 Department of Gastroenterology, Juntendo University Nerima Hospital, 3-1-10 Takanodai, Nerima-Ku, Tokyo 177-8521, Japan; 2 Division of Gastroenterology and Hepatology, Department of Internal Medicine (Omori), School of Medicine, Faculty of Medicine, Toho University, Omorinishi 6-11-1, Ota-Ku, Tokyo 143-8541, Japan; teppei.matsui@med.toho-u.ac.jp (T.M.); hidenari@med.toho-u.ac.jp (H.N.); 3 Department of Gastroenterology, Yokohama Hodogaya Central Hospital, 43-1 Kamadai-Cho, Hodogaya-Ku, Yokohama-Shi 240-8585, Japan; chuma@yokohama-cu.ac.jp; 4 Gastroenterological Center, Yokohama City University Medical Center, 4-57, Urafune-Cho, Minami-Ku, Yokohama-Shi 232-0024, Japan; t206071f@yokohama-cu.ac.jp (A.F.); morimoto.man.vx@yokohama-cu.ac.jp (M.M.); 5 Department of Gastroenterology and Hepatology, Faculty of Life Sciences, Kumamoto University, 1-1-1 Honjo, Chuo-Ku, Kumamoto 860-8556, Japan; kiruha@kumamoto-u.ac.jp (H.U.); narahara.satoshi@kuh.kumamoto-u.ac.jp (S.N.); ytanaka@kumamoto-u.ac.jp (Y.T.); 6 Gastroenterology Medicine Center, Shonan Kamakura General Hospital, 1370-1 Okamoto, Kamakura-City 247-8533, Japan; m_kobayashi4@shonankamakura.or.jp; 7 Department of Gastroenterology, St. Marianna University School of Medicine, 2-16-1 Sugao, Miyamae-Ku, Kawasaki-Shi 216-8511, Japan; yuwa.ando@marianna-u.ac.jp (Y.A.); twatanab@marianna-u.ac.jp (T.W.); 8 Department of Gastroenterology, Kanagawa Cancer Center, 2-3-2 Nakao, Asahi-Ku, Yokohama-Shi 241-8515, Japan; fukushima.0580h@kanagawa-pho.jp (T.F.); 9 Department of Gastroenterology, Shonan Tobu General Hospital, 500 Nishikubo, Chigasaki-Shi 253-0083, Japan; khirano-tym@umin.ac.jp; 10 Division of Gastroenterology and Hepatology, Department of Internal Medicine, Tokai University School of Medicine, 143 Shimokasuya, Isehara-Shi 259-1193, Japan; ktsuruya@tokai.ac.jp (K.T.); kagawa@tokai.ac.jp (T.K.); 11 Department of Gastroenterology, Internal Medicine, Kitasato University School of Medicine, 1-15-1 Kitasato, Minami-Ku, Sagamihara-Shi 252-0375, Japan; shu1rou@kitasato-u.ac.jp (S.I.); hisashi7@kitasato-u.ac.jp (H.H.); 12 Department of Gastroenterology, Yokohama City University Hospital, 3-9 Fukuura, Kanazawa-Ku, Yokohama-Shi 236-004, Japan; smaeda@yokohama-cu.ac.jp

**Keywords:** anti-programmed cell death 1, CRAFITY score, cytotoxic T-lymphocyte-associated protein 4, immune-related adverse event, immunotherapy selection, response rate

## Abstract

**Background/Objectives**: To investigate early treatment outcomes, including tumor response, predictors of response, and safety of nivolumab plus ipilimumab (Nivo/Ipi) therapy for unresectable hepatocellular carcinoma (uHCC) in a multicenter setting. **Methods**: We conducted a retrospective analysis of 64 Japanese patients with uHCC who received Nivo/Ipi therapy at participating centers between July 2025 and April 2026. Treatment efficacy was assessed according to RECIST version 1.1, and adverse events (AEs) were evaluated using CTCAE version 5.0. **Results**: Among the 64 patients, 28 received first-line therapy, and 36 received later-line therapy. The median observation period was 5.4 months. The overall response rate (ORR) was significantly higher in the first-line group (60.7%) than in the later-line group (30.6%) (*p* = 0.016). ORRs according to CRAFITY score were 25.0% for score 0, 55.6% for score 1, and 53.8% for score 2. The ORR in patients with CRAFITY scores ≥ 1 was significantly higher than in patients with a CRAFITY score of 0 (55.0% vs. 25.0%; *p* = 0.022). Multivariable analysis of pretreatment variables predicting response revealed first-line treatment (OR 3.83, 95% CI 1.270–11.558; *p* = 0.017) and a CRAFITY score ≥ 1 (OR 4.02, 95% CI 1.232–13.106; *p* = 0.021) as significant independent factors. Immune-mediated AEs occurred in 70.3% of patients overall and were grade ≥ 3 in 35.9%. **Conclusions**: Nivo/Ipi yielded a favorable response when used as first-line therapy. The baseline CRAFITY score may be a useful predictor of response to Nivo/Ipi therapy.

## Simple Summary

Nivolumab plus ipilimumab (Nivo/Ipi) is an emerging treatment option for unresectable hepatocellular carcinoma (uHCC); however, real-world data on its use in clinical practice remain limited. Because Nivo/Ipi is associated with a high incidence of adverse events, predictive factors that may help guide the selection of Nivo/Ipi in patients with uHCC are urgently needed. We conducted a retrospective multicenter analysis of 64 patients with uHCC who received Nivo/Ipi, evaluating tumor response according to RECIST version 1.1. First-line nivolumab plus ipilimumab was associated with a significantly higher response rate than later-line treatment. Additionally, a CRAFITY score of 1 or 2, which likely represents more aggressive and advanced malignancies, was identified as an independent predictor of tumor responses. The present study suggests that CRAFITY score may serve as a useful predictor of response to Nivo/Ipi therapy in routine clinical practice.

## 1. Introduction

Hepatocellular carcinoma (HCC) is the sixth most common cancer worldwide [1]. The treatment strategy for unresectable HCC (uHCC) has changed drastically following the advent of immune checkpoint inhibitors (ICIs). The IMbrave150 trial showed that the programmed cell death ligand 1 (PD-L1) inhibitor atezolizumab plus the vascular endothelial growth factor (VEGF) inhibitor bevacizumab (Atez/Bev) led to better overall survival (OS) and progression-free survival (PFS) than sorafenib [2]. The HIMALAYA trial showed that durvalumab (an anti-PD-L1 inhibitor) plus tremelimumab (an anti-cytotoxic T-lymphocyte-associated protein 4 (CTLA-4) antibody) (Dur/Tre) increased OS compared with sorafenib [3]. Current guidelines for first-line systemic therapy for uHCC recommend combination therapy with a single ICI or dual ICIs [4,5,6].

The CheckMate 9DW trial demonstrated that nivolumab (anti-programmed cell death 1 (PD-1) antibody) plus ipilimumab (anti-CTLA-4 antibody) (Nivo/Ipi) was superior to sorafenib or lenvatinib, a tyrosine kinase inhibitor (TKI), in terms of OS for uHCC [7]. Although a few studies have focused on the efficacy and safety of Nivo/Ipi in patients with uHCC who were previously exposed to ICIs [8,9], reports including predictive factors for response to Nivo/Ipi remain scarce. The present study aimed to investigate tumor response, predictors of response, and safety of Nivo/Ipi for uHCC in a real-world setting.

## 2. Materials and Methods

### 2.1. Patients

In this multicenter retrospective observational study, we included data from 64 Japanese patients with uHCC who were treated with Nivo/Ipi between July 2025 and April 2026 at 11 participating facilities: Juntendo University Nerima Hospital (*n* = 16), Toho University Omori Hospital (*n* = 10), Yokohama City Medical Center (*n* = 9), Kumamoto University Hospital (*n* = 6), St. Marianna University School of Medicine Hospital (*n* = 5), Shonan Kamakura General Hospital (*n* = 5), Kitasato University Hospital (*n* = 5), Kanagawa Cancer Center (*n* = 4), Yokohama Hodogaya Central Hospital (*n* = 2), Shonan Tobu General Hospital (*n* = 1) and Tokai University Hospital (n = 1). All patients received at least one cycle of Nivo/Ipi. Data from 3 patients who did not undergo radiological assessment were not included in the study. This study was approved by the Ethics Committee of Yokohama City University (approval No. 105, F25100033) and was conducted in accordance with the ethical standards outlined in the Declaration of Helsinki of 1964 and its later amendments. Informed consent was obtained from all the patients who participated in the study. The diagnosis of HCC was made based on high levels of alpha-fetoprotein (AFP) and imaging modalities such as abdominal dynamic computed tomography (CT), magnetic resonance imaging, and contrast-enhanced ultrasonography with perflubutane, or pre-treatment pathological findings. The most suitable treatment modality for each patient was determined through discussions among surgeons, hepatologists, and radiologists at each institution, in accordance with the Japanese practice guidelines for HCC [5]. The diagnosis of uHCC was made with caution after discussions among the doctors. Specifically, cases in which hepatic resection was contraindicated due to impaired cardiopulmonary function or in which ablation therapy was unfeasible due to the anatomical location of the tumor were considered unresectable HCC, even if the tumor was classified as BCLC stage A. The Common Terminology Criteria for Adverse Events (CTCAE) version 5.0 was used to assess AEs. In this study, immune-mediated AE (imAE) was assessed by the attending physician The therapeutic response was assessed by enhanced CT that was obtained 3–6 weeks after starting treatment and every 8–12 weeks subsequently, in accordance with the Response Evaluation Criteria in Solid Tumors (RECIST) version 1.1.

### 2.2. Treatment Protocol

Patients received intravenous administration of Nivo (80 mg/body) and Ipi (3 mg/kg) once every 3 weeks for up to 4 cycles, followed by 480 mg of nivolumab monotherapy every 4 weeks. Patients were required to attend weekly visits for at least the first six weeks after the initiation of Nivo/Ipi therapy. Treatment was interrupted at the discretion of the attending physician.

### 2.3. Statistical Analysis

Continuous variables are presented as median values with interquartile ranges (IQRs) and were analyzed using the Mann–Whitney U test. Categorical data were evaluated using the chi-square or Fisher’s exact test. PFS for each patient was defined as the time from the initiation of Nivo/Ipi treatment until disease progression, death, or the follow-up hospital visit. Similarly, overall survival OS was measured from the start of Nivo/Ipi treatment until death or the latest hospital visit date. PFS and OS were evaluated using the Kaplan–Meier method, and differences between the two groups were assessed using the log-rank test. Variables associated with treatment response with a *p*-value < 0.05 in the univariable analysis were further analyzed using multiple logistic regression analysis with the forced-entry method. All tests were two-sided, with significance set at *p* < 0.05. Statistical analyses were performed using SPSS Statistics for Windows (version 27; IBM Corp., Tokyo, Japan).

## 3. Results

### 3.1. Baseline Characteristics

The baseline characteristics of all patients are shown in Table 1. Among the 64 patients, 11 (17.2%) had a Child–Pugh score of ≥7, 36 (56.3%) received later-line therapy, and among the 18 patients with major vascular invasion, 5 had Vp4. Comparing the background characteristics of the first- and later-line groups, the Child–Pugh score was higher in the later-line group than in the first-line group (*p* = 0.005). Additionally, the observation period in the later-line group was significantly longer in the first-line group (6.1 vs. 4.0 months; *p* = 0.012).

### 3.2. Therapeutic Response

The overall responses according to treatment line are listed in Table 2. In the entire cohort, the overall response rate (ORR) was 43.8%, whereas the disease control rate (DCR) was 64.1%. The ORR was significantly higher in the first-line group than in the later-line group (60.7% vs. 30.6%, *p* = 0.016), whereas the DCR tended to be higher in the first-line group than in the later-line group (78.6% vs. 52.8%, *p* = 0.067). There were no significant differences in ORR (*p* = 1.00) or DCR (*p* = 0.735) between patients with Child–Pugh class A and B (Table S1).

The median C-reactive protein (CRP) and AFP levels were numerically greater in the response group than in the non-response group (0.66 vs. 0.32 mg/dL, *p* = 0.106; 345.1 vs. 79.4 ng/mL, *p* = 0.161, respectively). Therefore, we stratified the cohort according to the CRP and AFP in immunotherapy (CRAFITY) score, as described previously [10,11]. Specifically, scores of 0 (AFP < 100 ng/mL and CRP < 1.0 mg/dL), 1 (either AFP ≥ 100 ng/mL or CRP ≥ 1 mg/mL), or 2 (AFP ≥ 100 ng/mL and CRP ≥ 1 mg/mL) were assigned to each patient. The ORR increased from 25.0% in patients with a CRAFITY score of 0 to 55.6% in those with a score of 1, and 53.8% for those with a score of 2 (Table 3). After combining CRAFITY scores 1 and 2, the baseline characteristics according to CRAFITY score 0 and CRAFITY score ≥ 1 are listed in Table S2. Patients with a CRAFITY score ≥ 1 had a much higher rate of major vascular invasion, received Nivo/Ipi treatment at later lines, had lower serum albumin levels, and had higher des-γ-carboxy prothrombin (DCP) levels than those with a CRAFITY score of 0 (*p* = 0.044, *p* = 0.033, *p* = 0.014, and *p* = 0.026, respectively).

The ORR in patients with CRAFITY scores ≥ 1 was significantly higher than in patients with a CRAFITY score of 0 (55.0% vs. 25.0%; *p* = 0.043). In the analysis of only first-line patients, those with CRAFITY scores ≥ 1 had an ORR of 77.8%, while those with a score of 0 had an ORR of 30% (*p* = 0.02).

### 3.3. Univariable and Multivariable Analyses of Pretreatment Factors Related to the Therapeutic Response

Univariable and multivariable logistic regression analyses of baseline factors predicting the therapeutic response to Nivo/Ipi were conducted as shown in Table 4. Univariable analysis indicated that first-line treatment and CRAFITY score ≥ 1 were significantly related to the therapeutic response to Nivo/Ipi. Both first-line treatment (OR 3.832, 95% confidence interval (CI) 1.270–11.558; *p* = 0.017) and a CRAFITY score ≥ 1 (OR 4.019, 95% CI 1.232–13.106; *p* = 0.021) were identified as significant independent predictors of response in the multivariable analysis.

### 3.4. Progression Free Survivals

**Figure 1:**
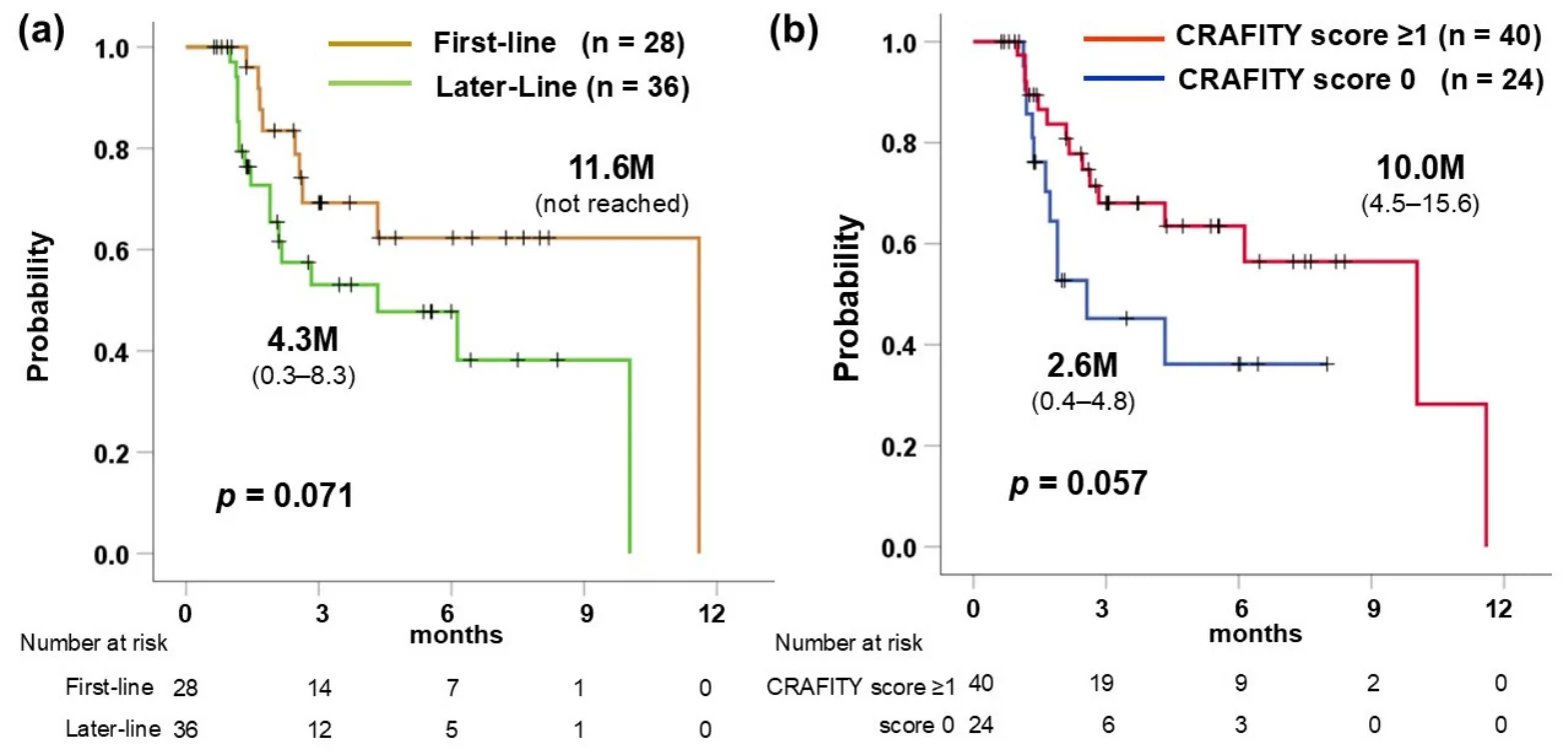
Kaplan–Meier curves for progression-free survival (PFS) according to (**a**) treatment line and (**b**) CRP and AFP in immunotherapy (CRAFITY) score.

Kaplan–Meier curves for PFS according to treatment line and CRAFITY score are shown in Figure 1. The median PFS of all 64 patients was 6.1 months. Median PFS in the first-line group was 11.6 months [95% CI not reached]), while that in the later-line group was 4.3 months ([95% CI 0.3–8.3], *p* = 0.071; Figure 1a). The median PFS in the CRAFITY score ≥ 1 (10.0 months [95% CI 4.5–15.6]) tended to be longer than that in the CRAFITY score 0 (2.6 months [95% CI 0.4–4.8], *p* = 0.057; Figure 1b). The median OS was not reached in the analysis according to the treatment line or CRAFITY score.

### 3.5. Safety

The rates of imAEs among the 64 patients who received Nivo/Ipi treatment are listed in Table 5. Any-grade and grade ≥ 3 imAEs occurred in 70.3% and 35.9% of patients, respectively. The most common any-grade imAEs were increased alanine aminotransferase (ALT) and aspartate aminotransferase (AST), both at 39.1%. Rash/pruritus followed in 28.2%, and colitis occurred in 18.8%. Additionally, grade ≥ 3 imAEs included increased AST (17.2%), increased ALT (15.6%), colitis (7.8%), type 1 diabetes mellitus (4.7%), rash/pruritus (3.1%), intestinal pneumonia (3.1%), hypoadrenocorticism (3.1%) and pancreatitis (3.1%). Thirty patients (47%) required steroid treatment, with a median prednisolone dose of 60 mg/day. Additionally, 22 patients (34.3%) received high-dose steroid treatment with ≥40 mg of prednisolone. In a cohort of 23 patients with grade ≥ 3 imAEs, three patients experienced grade 5 imAEs (one with interstitial pneumonia and two with hepatitis). The other 20 patients mainly improved with steroid treatment.

The median duration until the onset of imAEs among all patients was 28 days (95% CI 25–39) following the initiation of Nivo/Ipi. For patients undergoing first-line therapy, this period was 22 days (95% CI 17–39), whereas for those receiving later-line therapy, it was 33 days (95% CI 26–45) (*p* = 0.175). The incidence of imAE between first-line and later-line groups is listed in Table S3. There were no significant differences in the rate of any-grade and grade ≥ 3 imAEs between the first-line and later-line groups (*p* = 0.585 and *p* = 0.309, respectively).

The incidence of any-grade imAE hepatitis tended to be higher in patients with Child–Pugh class A than in those with Child–Pugh class B (*p* = 0.066), although this difference was not statistically significant (Table S4). Furthermore, no significant difference in the incidence of grade ≥ 3 imAE hepatitis was observed between the two groups (*p* = 0.672).

### 3.6. Predictive Factors for the Therapeutic Response After Nivo/ipi Treatment

**Figure 2:**
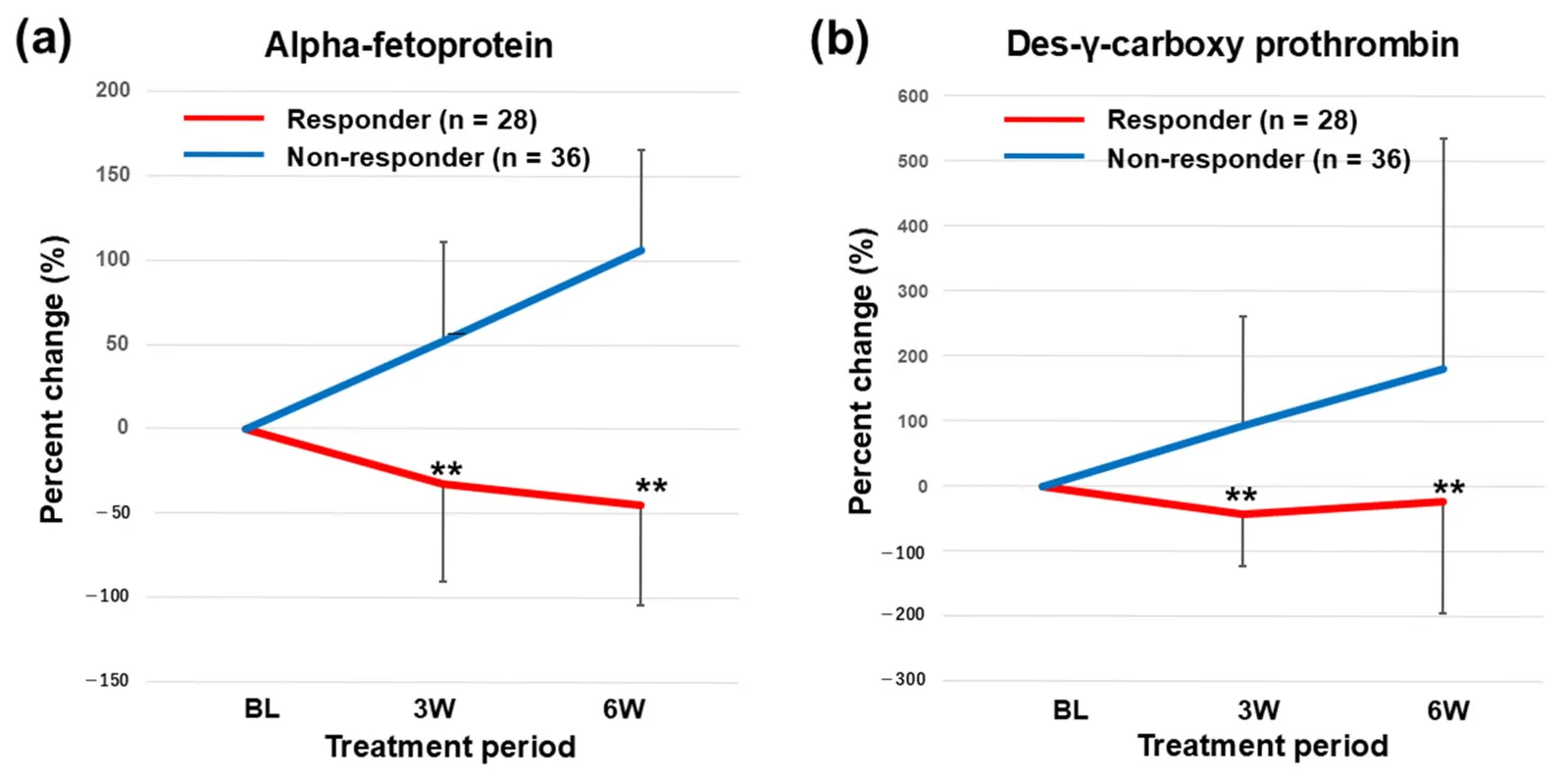
Time course of percent changes in (**a**) alpha-fetoprotein (AFP) and (**b**) des-γ-carboxy prothrombin (DCP) based on best overall response. ** *p* < 0.001 between the two groups.

Time course changes in tumor markers according to response are shown in Figure 2. The average percentage changes in AFP levels at 3 and 6 weeks were −32.3% and −44.8% for patients classified as responders and 52.4% and 106.3% for those classified as non-responders, respectively. For des-γ-carboxy prothrombin (DCP) levels, the average changes at 3 and 6 weeks were −41.7% and −22.6% for responders and 93.5% and 181.1% for non-responders, respectively. The median percentage changes in AFP and DCP at 3 and 6 weeks were significantly lower in responders than in non-responders (*p* < 0.001 for all comparisons). A receiver operating characteristic (ROC) curve analysis was conducted to evaluate changes in tumor markers for predicting tumor response (Figure S1). ROC analysis identified optimal cut-off values to predict tumor response of −4.439% for AFP with an area under the ROC curve (AUROC) of 0.785 and −8.998% for DCP (AUROC, 790) (Table 6).

Table 7 shows the ORR and DCR according to the occurrence of imAEs. The ORR (51.1% vs. 26.3%, *p* = 0.099) tended to be higher in patients who developed any-grade imAE than in those who did not. The DCR (71.1% vs. 47.4%, *p* = 0.025) was significantly higher in patients who developed any-grade imAE than in those who did not. There were no significant differences in either ORR (*p* = 0.974) or DCR (*p* = 0.591) between patients with and without grade ≥ 3 imAEs.

### 3.7. Relationship Between the Prior Treatment and Tumor Response with Nivo/ipi

We examined the relationship between prior systemic therapy and response to Nivo/Ipi in the 36 patients who received Nivo/Ipi as later-line therapy (Table 8). The ORR of Nivo/Ipi according to the last regimens, such as Atez/Bev, Dur/Tre, and TKI (lenvatinib), was 17.6% (3/17), 33.3% (2/6), and 42.5% (5/11), respectively.

### 3.8. Subsequent Therapy Following Nivo/ipi

We focused on the subsequent treatments administered to 43 patients who discontinued Nivo/Ipi therapy. A total of 20 (31.3%) patients ceased Nivo/Ipi therapy because of imAEs, whereas 21 (32.8%) discontinued because of progressive disease (PD). Of the 20 patients who discontinued treatment because of imAEs, 8 proceeded to subsequent treatment, including 4 who received systemic therapy. Similarly, of the 21 patients who discontinued treatment because of PD, 13 proceeded to subsequent treatment, with 9 receiving systemic therapy. Thus, the overall rate of transition to subsequent treatment following discontinuation because of either imAEs or PD was 21/43 (51.2%), with a 13/43 (30.2%) transition rate to systemic therapy.

## 4. Discussion

The multivariable analysis conducted in the present study identified two predictors of response to Nivo/Ipi: use as a first-line therapy and a CRAFITY score ≥ 1. These findings may help identify patients who are more likely to respond to Nivo/Ipi in routine clinical practice.

We found that the ORR was 60.7% with Nivo/Ipi used as first-line therapy. In the CheckMate 9DW trial, the ORR was 36% in the overall cohort and 56% in the Japanese cohort [7,12]. There are limited reports of real-world data for Nivo/Ipi in uHCC [8,9]. A retrospective analysis comparing ICI-naïve cases with cases previously treated with Atez/Bev revealed that the ORR was 20% and DCR was 49.1% in the Atez/Bev experienced cases [9]. In the present study, the ORR was 30.6% with Nivo/Ipi used as later-line therapy, indicating favorable outcomes. These findings suggest that Nivo/Ipi can also be considered a later-line therapy for uHCC.

The present study revealed a CRAFITY score ≥ 1 as an independent predictive factor for ORR in the multivariable analysis. Because Nivo/Ipi is associated with a high incidence of imAEs, careful consideration of the balance between efficacy and safety when selecting this regimen is necessary. Previous meta-analyses have shown that higher CRAFITY scores were associated with shorter OS and PFS, as well as lower ORR in uHCC treated with ICI excluding dual ICIs [13,14]. Ueno et al. reported that in Atez/Bev therapy for uHCC, a CRAFITY score of 0 was associated with a high ORR [11]. Conversely, Hatanaka et al. reported no association between the CRAFITY score and ORR in Atez/Bev therapy [15]. In the present study using Nivo/Ipi, the ORR for patients with a CRAFITY score ≥ 1was significantly higher than that for patients with a CRAFITY score of 0. CRP is a recognized indicator of cancer-related inflammation and is linked to cancer cell proliferation, angiogenesis, and suppression of adaptive immunity [16]. AFP is the most commonly accepted serological tumor marker for HCC and has been implicated in tumor proliferation, angiogenesis, and suppression of antitumor immune responses [17]. Our findings show an ORR of 77.8% among patients who received Nivo/Ipi as first-line therapy and had a CRAFITY score ≥ 1. A recent investigation into the gross classification and biological mechanisms of Atez/Bev treatment for uHCC demonstrated that the infiltrative subtype shows reduced efficacy to Atez/Bev, with regulatory T cell (Treg) accumulation in these tumors [18]. Anti-CTLA-4 antibodies, which induce antibody-dependent cellular cytotoxicity (ADCC) against intratumoral Tregs, serve as agents that decrease Treg levels [19]. Ipi is regarded as possessing more potent ADCC activity than Tre [20]. Tumors with high CRAFITY scores may exhibit significant accumulation of Tregs. Consequently, Nivo/Ipi may exert an antitumor effect in patients with a CRAFITY score ≥ 1. Because tumors with a high CRAFITY score could reflect advanced and/or aggressive characteristics, the durability of the response and its effect on long-term prognosis remain uncertain. Although this study focused on ORR as the initial treatment outcome, it was demonstrated that patients with a CRAFITY score of ≥1 exhibited a tendency for longer PFS than those with a score of 0. Further long-term follow-up and analyses with a larger sample size are warranted to assess the association of the CRAFITY score with durable response, PFS and OS.

HCC patients receiving ICIs have a higher risk of liver injury compared with those with non-HCC cancers treated with Nivo/Ipi [21]. When using Nivo/Ipi therapy for uHCC, extra care is needed because of the risk of imAE hepatitis compared with its use in other cancers. The CheckMate 9DW trial showed that the rates of any-grade and grade ≥ 3 imAE hepatitis in the Nivo/Ipi group were 14% and 6%, respectively [7]. In the present study, the rate of any-grade and grade ≥ 3 imAE hepatitis was 39% and 17%, respectively. This difference may be attributable, at least in part, to the inclusion of patients with Child–Pugh class B liver function, who represented 17% of the study population.

Both AFP and DCP are valuable biomarkers for assessing treatment response and prognosis in patients with HCC. Regarding the Dur/Tre regimen, both AFP and DCP are considered useful predictive factors for treatment response [22,23,24]. Uchikawa et al. identified that the optimal cut-off values to classify responders and non-responders at 4 weeks after initiation of Dur/Tre were −40% for AFP and −40.1% for DCP [22]. In the present study, the optimal cut-off values at three weeks were −4.439% for AFP and −8.998% for DCP. This difference may be attributable to differences in antitumor activity between Dur/Tre and Nivo/Ipi. Taken together, our findings suggest that relatively small changes in AFP and DCP at 3 weeks are helpful for identifying patients likely to respond to Nivo/Ipi therapy.

A meta-analysis demonstrated a positive correlation between the development of imAEs and ORR, PFS, and OS during ICI therapy, regardless of disease site and type of ICI [25]. No correlation was identified between the incidence of any-grade or grade ≥ 3 imAEs and the response rate in our real-world data of uHCC treated with Nivo/Ipi.

We also considered the use of Nivo/Ipi as part of sequential therapy and investigated the relationship between the therapeutic effect of Nivo/Ipi and both the previous regimen and the last regimen. Notably, the response to Nivo/Ipi may be influenced by the regimen immediately before Nivo/Ipi in sequential therapy, although this was not analyzed statistically because of the relatively small number of cases. Specifically, when Nivo/Ipi followed the administration of Atez/Bev, the ORR was relatively low at 17.6%. Recent studies have demonstrated that treatment outcomes with Dur/Tre following Atez/Bev are suboptimal [26,27]. Following Atez/Bev treatment, dual ICI may encounter challenges in attaining favorable efficacy. There is a paucity of reports on sequential therapy using dual ICI regimens. In our six cases involving the use of Nivo/Ipi following Dur/Tre, the ORR was 33.3%. The observed efficacy of Nivo/Ipi soon after Dur/Tre provides valuable real-world data regarding this sequential treatment strategy. The present study found that the ORR was 42.5% when lenvatinib, a TKI, was given before Nivo/Ipi. Among the response cases to Nivo/Ipi following prior Atez/Bev, 10 out of 11 patients had used a TKI immediately beforehand [9]. These findings, together with our data, suggest that the therapeutic sequence of Nivo/Ipi soon after a TKI may be an effective treatment sequence.

We found that the overall transition rate to subsequent treatment after discontinuation of Nivo/Ipi because of PD or severe imAEs was 51.2%. Reported rates of subsequent anticancer treatment after Dur/Tre treatment ranged from 63.8% to 94.7% [26,27]. This somewhat lower transition rate observed in the present study compared with that in the Dur/Tre reports may be attributable to the severity of imAEs associated with Nivo/Ipi, as well as the high proportion (56.2%) of later-line cases in the present study. This study demonstrated that subsequent treatment can be administered in a substantial proportion of patients after Nivo/Ipi treatment.

Our study has several limitations. First, the study was retrospective in nature and therefore subject to potential selection bias. Second, the study included a comparatively limited number of patients and a shorter follow-up duration. A larger cohort with a longer follow-up would be needed. Third, the analysis primarily focused on ORR. Previous reports suggested that tumor response could be used as a surrogate endpoint for OS to indicate which patients would be more likely to experience long-term benefits from systemic therapies for uHCC [28,29]. Therefore, despite these limitations, the present study provides clinically relevant evidence that the CRAFITY score may be useful as a predictor of response in patients receiving Nivo/Ipi.

## 5. Conclusions

In conclusion, our findings suggest that Nivo/Ipi yielded a favorable response when used as first-line therapy. The baseline CRAFITY score may be a useful predictor of response to Nivo/Ipi therapy, at least in short-term analyses. Nevertheless, larger-scale and longer-term follow-up studies are warranted.

## Figures and Tables

**Table 1:** Comparison in baseline characteristics between first-line and later line treatment group.

Variables	All	First-Line	Later-Line	*p*-Value
	*N* = 64	*n* = 28	*n* = 36	
Age, years, median (IQR)	68 (62–76)	69 (66–75)	66 (61–76)	0.485
Sex, male/female	55/9	25/3	30/6	0.72
Performance status ECOG: 0/1/2	61/3/0	26/2/0	35/1/0	0.577
Etiology, MASLD/ALD/HCV/HBV/others	22/16/13/6/7	9/9/6/1/3	16/9/4/3/4	0.673
BCLC Stage: A/B/C	1/23/40	1/11/16	0/12/24	0.927
Extrahepatic spread, yes/no	28/36	9/19	19/17	0.13
Major vascular invasion, yes/no	18/46	8/20	10/26	0.272
Treatment line: 1/2/3/4/5	28/16/14/5/1	28/0/0/0/0	0/16/14/5/1	<0.001
Child–Pugh score: 5/6/7/8/9	29/24/6/4/1	19/5/1/3/0	10/19/5/1/1	0.005
mALBI grade: 1/2a/2b/3	19/14/27/4	13/5/9/1	6/9/18/13	0.078
Total bilirubin, mg/dL, median (IQR)	0.9 (0.6–1.2)	0.9 (0.6–1.2)	0.9 (0.5–1.2)	0.905
Albumin, g/dL, median (IQR)	3.6 (3.2–4.0)	3.8 (3.2–4.2)	3.5 (3.2–3.8)	0.041
Aspartate aminotransferase, U/L, median (IQR)	43 (29–59)	43 (27–66)	44 (29–53)	0.876
Alanine aminotransferase, U/L, median (IQR)	29 (20–49)	31 (18–54)	29 (20–48)	0.725
Platelets, 10^4^/μL, median (IQR)	14.8 (10.3–22.5)	17.0 (11.3–26.9)	13.5 (9.8–20.5)	0.187
Prothrombin activity, %, median (IQR)	95 (82–105)	90 (82–103)	96 (82–103)	0.566
CRP, mg/mL, median (IQR)	0.38 (0.15–1.63)	0.5 (0.13–2.08)	0.38 (0.16–0.88)	0.641
AFP, ng/mL, median (IQR)	136 (8.4–2660)	71 (7.3–2290)	323 (8.4–2890)	0.561
DCP, mAU/mL, median (IQR)	579 (91–4017)	874 (59.5–4199)	579 (132–3890)	0.792
CRAFITY score: 0/1/2	24/27/13	10/13/5	14/14/8	0.818
NLR, median (IQR)	2.77 (2.25–4.65)	2.85 (1.74–4.55)	2.75 (2.43–5.07)	0.598
Observation period, month, median (IQR)	5.4 (2.9–8.1)	4.0 (2.2–9.6)	6.1 (3.3–9.6)	0.012

Abbreviations: AFP, α-fetoprotein; ALD, alcoholic liver disease; BCLC, Barcelona Clinic Liver Cancer; CRAFITY, C-reactive protein and AFP in immunotherapy; CRP, C-reactive protein, DCP, des-γ-carboxy prothrombin; HBV, hepatitis B virus; HCV, hepatitis C virus; IQR, interquartile range; mALBI, modified albumin–bilirubin; MASLD, metabolic dysfunction-associated steatotic liver diseases; NLR, neutrophil-to-lymphocyte ratio.

**Table 2:** Overall tumor response based on RECISTv1.1 according to treatment line.

		Treatment Line		
	All (*N* = 64)	First (*n* = 28)	Later (*n* = 36)	*p*-Value
CR, *n* (%)	2 (3.1)	1 (3.6)	1 (2.8)	
PR, *n* (%)	26 (40.6)	16 (57.1)	10 (27.8)	
SD, *n* (%)	13 (20.3)	5 (17.9)	8 (22.2)	
PD, *n* (%)	23 (35.9)	6 (21.4)	17 (47.2)	
ORR, %	43.8	60.7	30.6	0.016
DCR, %	64.1	78.6	52.8	0.067

Abbreviations: CR, complete response; DCR, disease control rate; ORR, overall response rate; PD, progressive disease; PR, partial response; SD, stable disease.

**Table 3:** Overall tumor response based on RECISTv1.1 stratified by CRAFITY score.

	All (*N* = 64)				First-Line (*n* = 28)				Later-Line (*n* = 36)			
**CRAFITY Score**	**0**	**1**	**2**	***p*** **-Value**	**0**	**1**	**2**	***p*** **-Value**	**0**	**1**	**2**	***p*** **-Value**
	***n* = 24**	***n* = 27**	***n* = 13**		***n* = 10**	***n* = 13**	***n* = 5**		***n* = 14**	***n* = 14**	***n* = 8**	
CR, *n*	0	1	1		0	1	0		0	0	1	
PR, *n*	6	14	6		3	9	4		3	5	2	
SD, *n*	7	5	1		3	1	1		4	4	0	
PD, *n*	11	7	5		4	2	0		7	5	5	
ORR, %	25.0	55.6	53.8	0.064	30.0	76.9	80.0	0.046	21.4	35.7	37.5	0.635
DCR, %	54.2	74.1	61.5	0.313	60.0	84.6	100	0.158	50.0	64.3	37.5	0.702
**CRAFITY score**	**0**	**1 or 2**		***p*** **-Value**	**0**	**1 or 2**		***p*** **-Value**	**0**	**1 or 2**		***p*** **-Value**
	***n* = 24**	***n* = 40**			***n* = 10**	***n* = 18**			***n* = 14**	***n* = 22**		
CR, *n*	0	2			0	1			0	1		
PR, *n*	6	20			3	13			3	7		
SD, *n*	7	6			3	2			4	4		
PD, *n*	11	12			4	2			7	10		
ORR, %	25.0	55.0		0.022	30.0	77.8		0.020	21.4	36.4		0.467
DCR, %	54.2	70.0		0.135	60.0	88.9		0.147	50.0	54.5		0.734

Abbreviations: CR, complete response; CRAFITY, CRP and AFP in immunotherapy; DCR, disease control rate, ORR, overall response rate; PD, progressive disease; PR, partial response; SD, stable disease.

**Table 4:** Univariable and multivariable analyses of pretreatment factors related to the therapeutic response to nivolumab and ipilimumab.

	Univariate Analysis		Multivariate Analysis	
Variable	OR (95% CI)	*p*-Value	OR (95% CI)	*p*-Value
Age, ≥70	0.891 (0.327–2.424)	0.821		
Sex, male	0.968 (0.234–3.998)	0.964		
Etiology, viral	1.381 (0.421–4.533)	0.595		
mALBI 1 or 2a	1.154 (0.429–3.103)	0.777		
Extrahepatic spread	0.556 (0.202–1.529)	0.255		
Major vascular invasion	1.421 (0.476–4.246)	0.529		
BCLC stage C	0.667 (0.240–1.899)	0.436		
Treatment line, first	3.512 (1.244–9.920)	0.018	3.832 (1.270–11.558)	0.017
CRAFITY score, ≥1	3.667 (1.203–11.174)	0.022	4.019 (1.232–13.106)	0.021
DCP, ≥1000 mAU/mL	0.969 (0.360–2.606)	0.95		
NLR, ≤2.74	1.490 (0.551–4.027)	0.432		

Abbreviations: BCLC, Barcelona Clinic Liver Cancer; CI, confidence interval; CRAFITY, C-reactive protein and AFP in immunotherapy; DCP, des-γ-carboxy prothrombin; NLR, neutrophil to lymphocyte ratio; OR, odds ratio.

**Table 5:** Immune-mediated adverse events during nivolumab plus ipilimumab treatment.

	ALL (*N* = 64)	
	Any-Grade; *n* (%)	Grade ≥ 3; *n* (%)
Any imAE	45 (70.3)	23 (35.9)
Increased AST	25 (39.1)	11 (17.2)
Increased ALT	25 (39.1)	10 (15.6)
Rash/pruritus	18 (28.2)	2 (3.1)
Colitis	12 (18.8)	5 (7.8)
Hypoadrenocorticism	7 (10.9)	2 (3.1)
Thyroid dysfunction	3 (4.7)	1 (1.6)
Intestinal pneumonia	4 (6.3)	2 (3.1)
Type 1 diabetes mellitus	3 (4.7)	3 (4.7)
Pancreatitis	2 (3.1)	2 (3.1)
Thrombocytopenia	2 (3.1)	1 (1.6)
Myocarditis	1 (1.6)	1 (1.6)
Neuropathy	2 (3.1)	0

Abbreviations: AST, aspartate aminotransferase; ALT, alanine aminotransferase; imAE, immune-mediated adverse event.

**Table 6:** ROC curve analysis of each parameter and prediction of tumor response.

	Cut-Off Value	AUROC	Sensitivity, %	Specificity, %	95% CI	*p*-Value
ΔAFP(%)_3w-BL_	−4.439	0.785	73.0	87.0	0.656–0.914	<0.001
ΔDCP(%)_3w-BL_	−8.998	0.79	83.0	68.0	0.671–0.908	<0.001

Abbreviations: Δ(%)_3w-BL_, percentage change from baseline to 3 weeks; AFP, alpha-fetoprotein; AUROC, area under the ROC curve; BL, baseline; CI, confidence interval, DCP, des-γ-carboxy prothrombin; ROC, receiver operating characteristic.

**Table 7:** Overall tumor response according to development of imAE.

	Any-Grade imAE			Grade ≥ 3 imAE		
	With	Without	*p*-Value	With	Without	*p*-Value
	*n* = 45	*n* = 19		*n* = 23	*n* = 41	
CR, *n* (%)	1 (2.2)	1 (5.3)		1 (4.4)	1 (2.4)	
PR, *n* (%)	22 (48.9)	4 (21.1)		9 (39.1)	17 (41.5)	
SD, *n* (%)	9 (20.0)	4 (21.1)		4 (17.4)	9 (22.0)	
PD, *n* (%)	13 (28.9)	10 (52.6)		9 (39.1)	14 (34.1)	
ORR, %	51.1	26.3	0.099	43.5	43.9	0.974
DCR, %	71.1	47.4	0.025	60.9	65.9	0.591

Abbreviations: CR, complete response; DCR, disease control rate; imAE, immune-mediated adverse event; ORR, overall response rate; PD, progressive disease; PR, partial response; SD, stable disease.

**Table 8:** Relationship between prior regimen and therapeutic response to nivolumab plus ililimumab.

Previous Use Regimen	Atezolizumab Plus Bevacizumab		Durvalumab Plus Tremelimumab		Tyrosine Kinase Inhibitor	
	Yes	No	Yes	No	Yes	No
	*n* = 33	*n* = 3	*n* = 8	*n* = 28	*n* = 14	*n* = 22
CR, *n*	1	0	1	0	0	1
PR, *n*	9	1	1	9	4	6
SD, *n*	8	0	2	6	3	5
PD, *n*	15	2	4	13	7	10
ORR, %	30.3	33.3	25.0	32.1	28.6	31.8
DCR, %	54.5	33.3	50.0	53.6	50.0	54.5
**The last regimen**	**Atezolizumab plus** **bevacizumab**		**Durvalumab plus tremelimumab**		**Tyrosine kinase inhibitor**	
	**Yes**	**No**	**Yes**	**No**	**Yes**	**No**
	***n*** **= 17**	***n* = 19**	***n* = 6**	***n* = 30**	***n* = 11**	***n* = 25**
CR, *n*	0	1	1	0	0	1
PR, *n*	3	7	1	9	5	5
SD, *n*	4	4	1	7	3	5
PD, *n*	10	7	3	14	3	14
ORR, %	17.6	42.1	33.3	30.0	45.5	25.0
DCR, %	41.2	63.1	50.0	53.3	72.7	44.0

Abbreviations: CR, complete response; DCR, disease control rate; ORR, overall response rate; PD, progressive disease; PR, partial response; SD, stable disease.

## Data Availability

Data are contained within the article and the Supplementary Materials.

## Supplementary Materials

The following supporting information can be downloaded at: https://www.mdpi.com/article/10.3390/cancers18162703/s1, Table S1. Overall tumor response based on RECISTv1.1 stratified by Child–Pugh classification; Table S2. Comparison in baseline characteristics between CRAFITY score 0 and CRAFITY score 1 or 2; Table S3. Comparison of occurrence of immune-mediated adverse events between first-line and later-line groups; Table S4. Comparison of occurrence of treatment-related liver injury stratified by Child–Pugh classification; Figure S1. Receiver operating characteristic (ROC) analysis of changes in tumor markers to predict overall response.

## Abbreviations

The following abbreviations are used in this manuscript:

ADCC	antibody-dependent cellular cytotoxicity
AE	adverse event;
AFP	alpha-fetoprotein
Atez	atezolizumab
AUROC	area under the receiver operating characteristics curve
BCLC	Barcelona Clinic Liver Cancer
Bev	bevacizumab
BL	baseline
CI	confidence interval
CRAFITY	CRP and AFP in immunotherapy
CRP	C-reactive protein
CT	computed tomography
CTCAE	Common Terminology Criteria for Adverse Events
CTLA-4	cytotoxic T-lymphocyte-associated protein 4
DCP	des-γ-carboxy prothrombin
DCR	disease control rate
Dur	durvalumab
eGFR	estimated glomerular filtration rate
HCC	hepatocellular carcinoma
ICI	immune checkpoint inhibitor
imAE	immune-mediated adverse event
Ipi	ipilimumab
mALBI	modified albumin–bilirubin
MASLD	metabolic dysfunction-associated steatotic liver diseases
Nivo	nivolumab
NLR	neutrophil-to-lymphocyte ratio
ORR	overall response rate
OS	overall survival
PD	progressive disease
PD-L1	programmed cell death ligand 1
PD-1	programmed cell death 1
PR	partial response
PS	performance status
RECIST	Response Evaluation Criteria in Solid Tumors
SD	stable disease
TKI	tyrosine kinase inhibitor
Treg	regulatory T cell
Tre	tremelimumab
uHCC	unresectable hepatocellular carcinoma
VEGF	vascular endothelial growth factor
