# Supplementary material for: Nivolumab Plus Ipilimumab for Unresectable Hepatocellular Carcinoma in Early Real-World Clinical Practice: A Multicenter Analysis of Efficacy and Safety
Source: Cancers (Basel). 2026 Aug 20;18(16):2703. doi: 10.3390/cancers18162703 (PMC13510368; doi:10.3390/cancers18162703)
Supplement: Supplementary file 1 [file cancers-18-02703-s001.zip › cancers-4492345-supplementary.pdf]

Table S1. Overall tumor response based on RECISTv1.1 stratified by Child-Pugh classification.

	**Child-Pugh class A** *n* **= 53**	**Child-Pugh class B** *n* **= 11**	***p*-Value**
CR, *n* (%)	2 (3.8)	0	
PR, *n* (%)	21 (39.6)	5 (45.5)	
SD, *n* (%)	10 (18.9)	3 (27.3)	
PD, *n* (%)	20 (37.7)	3 (27.3)	
ORR, %	43.4	45.5	1.000
DCR, %	62.3	72.7	0.735

Abbreviations: CR, complete response; DCR, disease control rate, ORR, overall response rate; PD, partial response; PR, partial response; SD, stable disease.

Table S2. Comparison in baseline characteristics between CRAFITY score 0 and CRAFITY score 1 or 2.

	**CRAFITY score 0** *n* **= 24**	**CRAFITY score ≥1** *n* **= 40**	***p*-Value**
Age, years, median (IQR)	68 (64–76)	69 (61–76)	0.792
Sex, male/female	21/3	34/6	1.000
Performance status ECOG: 0/1/2	24/0	37/3	0.286
Etiology, MASLD/ALD/HCV/HBV/others	10/4/2/4/4	12/12/11/2/3	0.103
BCLC Stage: A/B/C	0/11/13	1/12/27	0.355
Extrahepatic spread, yes/no	12/12	16/24	0.435
Major vascular invasion, yes/no	3/21	15/25	0.044
Treatment line: 1/2/3/4/5	10/10/3/0/1	18/6/11/5/0	0.033
Child-Pugh score: 5/6/7/8/9	15/5/2/2/0	14/19/4/2/1	0.181
mALBI grade: 1/2a/2b/3	9/6/9/0	10/8/18/4	0.313
Total bilirubin, mg/dL, median (IQR)	1.0 (0.6–1.2)	0.8 (0.5–1.2)	0.597
Albumin, g/dL, median (IQR)	3.8 (3.5–4.3)	3.5 (3.1–3.9)	0.014
Aspartate aminotransferase, U/L, median (IQR)	42 (29–53)	45 (28–76)	0.723
Alanine aminotransferase, U/L, median (IQR)	34 (23–47)	29 (17–50)	0.734
Platelets, 104/μL, median (IQR)	14.8 (9.9–20.5)	15.1 (10.4–23.8)	0.618
Prothrombin activity, %, median (IQR)	96 (87–103)	92 (78–109)	0.408
CRP, mg/mL, median (IQR)	0.18 (0.08–0.31)	0.84 (0.36–2.54)	<0.001
AFP, ng/mL, median (IQR)	6.8 (2.4–35.1)	664 (149–5512)	<0.001
DCP, mAU/mL, median (IQR)	149 (50–2810)	1412 (235–4199)	0.026
CRAFITY score: 0/1/2	24/0/0	0/27/13	<0.001
NLR, median (IQR)	2.73 (1.65–4.64)	2.86 (2.32–4.63)	0.255
Observation period, month, median (IQR)	4.8 (2.7–7.3)	5.4 (3.0–8.9)	0.398

Abbreviations: AFP, α-fetoprotein; ALD, alcoholic liver disease; BCLC, Barcelona Clinic Liver Cancer; CRAFITY, C-reactive protein and AFP in immunotherapy; CRP, C-reactive protein, DCP, des-γ-carboxy prothrombin; HBV, hepatitis B virus; HCV, hepatitis C virus; IQR, interquartile range; mALBI, modified albumin–bilirubin; MASLD, metabolic dysfunction-associated steatotic liver diseases; NLR, neutrophil-to-lymphocyte ratio.

Table S3. Comparison of occurrence of immune-mediated adverse events between first-line and later-line groups.

**Treatment line**	**First, *n* = 28**	**Later, *n* = 36**		**First, *n* = 28**	**Later, *n* = 36**	
	**Any grade; *n* (%)**		***p*-Value**	**Grade ≥3; *n* (%)**		***p*-Value**
Any imAE	21 (75)	24 (66.7)	0.585	12 (42.9)	11 (30.6)	0.309
Increased AST	12 (42.9)	13 (36.1)		6 (21.4)	5 (13.9)	
Increased ALT	12 (42.9)	13 (36.1)		6 (21.4)	4 (11.1)	
Rash/pruritus	9 (32.1)	9 (25)		1 (3.6)	1 (2.8)	
Colitis	4 (14.3)	8 (22.2)		3 (10.7)	2 (5.6)	
Hypoadrenocorticism	4 (14.3)	3 (8.3)		1 (3.6)	1 (2.8)	
Thyroid dysfunction	2 (7.1)	1 (2.8)		0	1 (2.8)	
Intestinal pneumonia	3 (10.7)	1 (2.8)		2 (7.1)	0	
Type 1 diabetes mellitus	3 (10.7)	0		3 (10.7)	0	
Pancreatitis	1 (3.6)	1 (2.8)		1 (3.6)	1 (2.8)	
Thrombocytopenia	1 (3.6)	1 (2.8)		1 (3.6)	0	
Myocarditis	0	1 (2.8)		0	1 (2.8)	
Neuropathy	0	2 (3.6)		0	2 (5.6)	

Abbreviations: AST, aspartate aminotransferase; ALT, alanine aminotransferase; imAE, immune-mediated adverse event.

Table S4. Comparison of occurrence of treatment-related liver injury stratified by Child-Pugh classification.

	Child-Pugh class A<br>*n* = 53	Child-Pugh class B<br>*n* = 11		Child-Pugh class A<br>*n* = 53	Child-Pugh class B<br>*n* = 11	
	Any-grade; *n* (%)		*p*-Value	Grade ≥3; *n* (%)		*p*-Value
Increased AST	18 (34.0)	7 (63.6)	0.066	10 (18.9)	1 (9.1)	0.672
Increased ALT	18 (34.0)	7 (63.6)	0.066	9 (17.0)	1 (9.1)	1.000

Abbreviations: AST, aspartate aminotransferase; ALT, alanine aminotransferase.

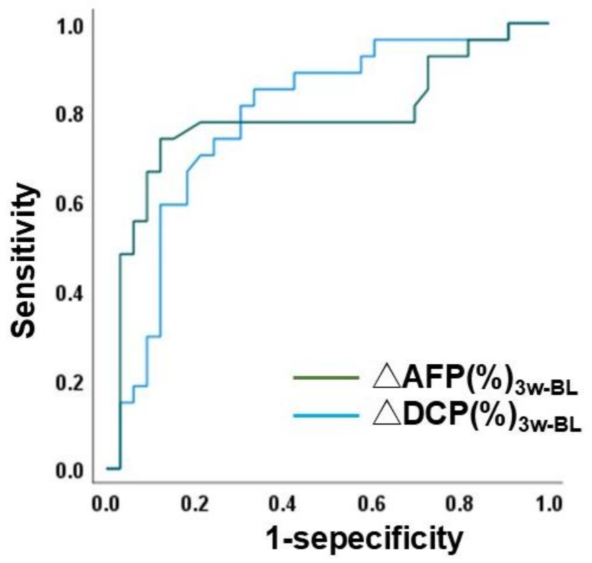

Figure S1. Receiver operating characteristic analysis of changes in tumor markers to predict overall response. Abbreviations: △(%)3w-BL, percentage change from baseline to 3 weeks; AFP, alpha-fetoprotein; DCP, des-γ-carboxy prothrombin.
